# Positive Allosteric Modulation of the Glucagon-like Peptide-1 Receptor by Diverse Electrophiles*

**DOI:** 10.1074/jbc.M115.696039

**Published:** 2016-03-14

**Authors:** Ana B. Bueno, Aaron D. Showalter, David B. Wainscott, Cynthia Stutsman, Aranzazu Marín, James Ficorilli, Over Cabrera, Francis S. Willard, Kyle W. Sloop

**Affiliations:** From the ‡ Centro de Investigación Lilly, Eli Lilly and Co., Alcobendas 28108, Spain and; § Endocrine Discovery and; ¶ Quantitative Biology, Lilly Research Laboratories, Eli Lilly and Co., Indianapolis, Indiana 46285

**Keywords:** allosteric regulation, diabetes, G protein-coupled receptor (GPCR), insulin secretion, pancreatic islet, glucagon-like peptide-1

## Abstract

Therapeutic intervention to activate the glucagon-like peptide-1 receptor (GLP-1R) enhances glucose-dependent insulin secretion and improves energy balance in patients with type 2 diabetes mellitus. Studies investigating mechanisms whereby peptide ligands activate GLP-1R have utilized mutagenesis, receptor chimeras, photo-affinity labeling, hydrogen-deuterium exchange, and crystallography of the ligand-binding ectodomain to establish receptor homology models. However, this has not enabled the design or discovery of drug-like non-peptide GLP-1R activators. Recently, studies investigating 4-(3-benzyloxyphenyl)-2-ethylsulfinyl-6-(trifluoromethyl)pyrimidine (BETP), a GLP-1R-positive allosteric modulator, determined that Cys-347 in the GLP-1R is required for positive allosteric modulator activity via covalent modification. To advance small molecule activation of the GLP-1R, we characterized the insulinotropic mechanism of BETP. In guanosine 5′-3-*O*-(thio)triphosphate binding and INS1 832-3 insulinoma cell cAMP assays, BETP enhanced GLP-1(9–36)-NH_2_-stimulated cAMP signaling. Using isolated pancreatic islets, BETP potentiated insulin secretion in a glucose-dependent manner that requires both the peptide ligand and GLP-1R. In studies of the covalent mechanism, PAGE fluorography showed labeling of GLP-1R in immunoprecipitation experiments from GLP-1R-expressing cells incubated with [^3^H]BETP. Furthermore, we investigated whether other reported GLP-1R activators and compounds identified from screening campaigns modulate GLP-1R by covalent modification. Similar to BETP, several molecules were found to enhance GLP-1R signaling in a Cys-347-dependent manner. These chemotypes are electrophiles that react with GSH, and LC/MS determined the cysteine adducts formed upon conjugation. Together, our results suggest covalent modification may be used to stabilize the GLP-1R in an active conformation. Moreover, the findings provide pharmacological guidance for the discovery and characterization of small molecule GLP-1R ligands as possible therapeutics.

## Introduction

The secretin family of peptide hormone-binding class B G protein-coupled receptors (GPCRs)^2^ is composed of 15 members that help regulate various physiological systems, including growth, the stress response, and glucose metabolism. These receptors possess a long amino-terminal ectodomain (ECD) that is structurally stabilized by three evolutionarily conserved disulfide bonds (1–3). This characteristic ECD is a globular structure hypothesized to be critical for the initial ligand recognition event. Subsequent interactions between the docked peptide ligand and the helical bundle of the receptor core occur to induce signaling (4). Several reports provide data supporting the initial receptor-ligand recognition mechanism, and among the most informative are the crystallography studies using the purified ECD regions in complex with their cognate ligands (5, 6) and a recent report utilizing electron microscopy and hydrogen-deuterium exchange study of peptide-ligated glucagon receptor (7). These studies clearly define many intermolecular processes utilized by various ECDs to bind with great specificity to the orthosteric peptide. However, crystal structures of ligands bound to full-length class B receptors are needed to fully determine critical interactions with the helical bundle that induce an active receptor conformation.

Although more than 20 class A GPCR crystal structures have been resolved to date, the resolution of class B GPCRs has been elusive. Recently, high resolution structures of the transmembrane domains of two class B family members, the glucagon receptor (8) and corticotropin-releasing factor receptor-1 (9), have been solved. Although the structures were determined using receptor constructs that did not include the ECD sequences, data from both predict very deep and open V-shaped binding crevices for the orthosteric ligands (10). Although several peptide-based agonist therapeutics have been developed for class B GPCRs (11), small molecule agonists have not been developed. One hypothesis for this intractability of class B GPCRs is that the intricate nature of receptor activation renders it challenging to identify low molecular weight non-peptide orthosteric ligands that make sufficient high affinity receptor contacts over large interaction surfaces to mimic the activation mechanism used by the peptide agonists. This may prove especially difficult if crystal structures of other receptor core domains are solved and show this deep crevice-like binding is general to class B GPCRs. Therefore, an alternative approach to enhance receptor activation is to identify small molecule positive allosteric modulators (PAMs) that bind topographically distinct sites and relieve some hindrance of transitioning the receptor to an active state. Several reports have described the identification and development of PAMs for class A, class B, and class C GPCRs (12).

The glucagon-like peptide-1 receptor (GLP-1R) is a class B GPCR that has proved to be an effective therapeutic target as several peptide-based agonists have been developed and registered for treating type 2 diabetes mellitus (13, 14). Reports from preclinical studies have described the discovery of small molecule GLP-1R PAMs (15, 16). Two of the most characterized are compound 2 (17, 18) and compound B/BETP (19); both molecules have been shown to potentiate GLP-1R-stimulated cAMP accumulation by oxyntomodulin (20, 21) and GLP-1(9–36)-NH_2_ (22, 23). Each compound also enhances the actions of non-natural agonists on the GLP-1R, peptidic and non-peptidic, such as BMS-21, Boc-5, and TT-15 (24). In total, these results support the existence of an allosteric site in the GLP-1R distinct from the orthosteric peptide-binding pocket. Although structurally distinct, unfortunately both compounds are unstable and display poor pharmacokinetic properties that preclude potential clinical development (16). Not surprisingly, because of its electrophilic nature, BETP has been reported to conjugate with GSH *in vitro* (25).

The finding that nucleophilic GSH displaces the ethyl sulfoxide moiety of BETP led to studies that elegantly demonstrated the allosteric mechanism utilized by BETP to potentiate GLP-1R signaling occurs via irreversible covalent modification of a free cysteine located in the third intracellular loop of the GLP-1R (26). Importantly, substitution of this cysteine (position 347) with alanine does not alter peptide agonism at the mutant receptor but does result in loss of PAM action for both BETP and compound 2 (26). Interestingly, although BETP and compound 2 represent distinct chemical pharmacophores, the compounds share the electrophilicity; this suggests that compound 2 also activates the GLP-1R by covalent modification of Cys-347 (26). Although both molecules act by the same mechanism, modification of the receptor by the compounds results in a differential enhancement of GLP-1R signaling (cAMP accumulation *versus* intracellular calcium mobilization, β-arrestin recruitment, and ERK phosphorylation) (24). Although BETP and compound 2 are the best characterized GLP-1R PAMs reported to date, other modulators have been described (16), but it is not known whether these molecules interact with the receptor by the same covalent mechanism. Furthermore, it is unclear whether other electrophiles that can access Cys-347 will modulate GLP-1R activity.

For class B GPCRs, the presence of a free cysteine in the GLP-1R at position 347 is unique. Thus, studies evaluating the effect of modifying this site on GLP-1R-mediated cellular function may enable the development of receptor-specific agents. In line with this, the work presented herein shows that BETP potentiates insulin secretion in a glucose- and GLP-1R-dependent manner, therefore bolstering the potential therapeutic utility of allosterically modulating receptor activity. To further interrogate the mechanism whereby covalent modification enhances GLP-1R function, we describe the discovery and characterization of several electrophilic chemotypes that potentiate GLP-1R activity in a Cys-347-dependent manner. Importantly, the results indicate that structurally diverse compounds that have the ability to access the third inner membrane loop of the GLP-1R can enhance receptor signaling of this important therapeutic target. Because the positive allosteric mechanism likely occurs through structural modification that results in the formation of an intracellular adduct, strategies aimed at targeting this site may facilitate efforts to stabilize the GLP-1R in an active confirmation and possibly enable new ligand identification approaches.

## Experimental Procedures

### Ligands

The following compounds were prepared as described previously: BETP and th-BETP (24); **1** and **2** (17, 18); **3** (27); **4** and **5** (28); **6** (29); **7** and **8** (30); **9** (27); **15** (31); **17** (32). Other described compounds were purchased from commercial sources. [^3^H]BETP was purchased from ViTrax (specific activity, 33.3 Ci/mmol). GLP-1(7–36)-NH_2_ was synthesized at Lilly Research Laboratories, and GLP-1(9–36)-NH_2_ was purchased from Bachem (Torrance, CA).

### GLP-1R [^35^S]GTPγS Binding Assays

Preparation of GLP-1R HEK293 cell membranes and measurement of GLP-1R activation via [^35^S]GTPγS binding to Gα_s_ using an antibody capture scintillation proximity assay were performed as described previously (20). Briefly, reactions contained 5 μg of membrane in 20 mm HEPES, pH 7.4, 50 mm NaCl, 5 mm MgCl_2_, 40 μg/ml saponin, 0.1% bovine serum albumin, and 500 pm ^35^S-labeled GTPγS (PerkinElmer Life Sciences). Peptides and compounds were diluted and treated at a final concentration of 1% DMSO. Binding was induced for 30 min at room temperature before solubilization with 0.2% Nonidet P-40 detergent, rabbit anti-Gα_s_ polyclonal antibody, and 0.5 mg of anti-rabbit polyvinyl toluene beads (PerkinElmer Life Sciences). The detection mixtures were developed for 30 min, centrifuged at 80 × *g* for 10 min, and counted for 1 min/well using a MicroBeta TriLux instrument (PerkinElmer Life Sciences).

### INS1 832-3 Cell cAMP Accumulation Assays

The INS1-derived 832-3 insulinoma cell line (33, 34) was used to study stimulation of intracellular cAMP by BETP + GLP-1(9–36)-NH_2_. Cells were maintained by growing adherently in RPMI 1640 medium (HyClone, Pittsburgh, PA) containing 11.2 mm glucose supplemented with 10% fetal bovine serum, 10 mm HEPES, 1 mm sodium pyruvate, 2 mm l-glutamine, 100 units/ml penicillin, 100 μg/ml streptomycin, and 50 μm 2-mercaptoethanol at 37 °C in 5% CO_2_. On the day of the assay, cells were lifted, counted, and resuspended in Earle's balanced salt solution buffer (EBSS) containing 0.1% bovine serum albumin and 11.2 mm glucose. Cells were seeded at a density of 40,000 cells per well into sterile 96-well half-area solid black microplates and incubated in the presence of various treatments supplemented with isobutylmethylxanthine at 37 °C for 30 min. Cells were then assayed for cAMP accumulation using homogeneous time-resolved fluorescence technology (Cisbio Bioassays, Bedford, MA).

### Ex Vivo Pancreatic Islet Insulin Secretion Assays

Male wild-type and *Glp-1r* null mice (35) (both of C57BL/6 background) were maintained in accordance with the Institutional Animal Care and Use Committee of Eli Lilly and Co. and the Guide for the Use and Care of Laboratory Animals by the National Institutes of Health. Mice were housed in microisolator cages on wood chip bedding with food (2014 Teklad Global Diet, Harlan, Indianapolis, IN) and deionized water available *ad libitum*. Lights were on a 12:12-h light/dark cycle, and temperature and relative humidity were maintained between 21 and 23 °C and 45 and 65%, respectively. For pancreatic islet isolation, mice were euthanized by cervical dislocation. The common bile duct was cannulated with a 27-gauge needle, and the pancreas was distended by infusion of 10 ml of Hanks' balanced salt solution buffer (Sigma) containing 2% bovine serum albumin (Akron Biotech, Boca Raton, FL) and 1 mg/ml collagenase (Vitacyte, Indianapolis, IN). Pancreatic tissue was then removed and digested in Hanks' balanced salt solution buffer at 37 °C. Islets were purified on a Histopaque (Histopaque-1077/Histopaque-11991 mixture) gradient (Sigma) for 18 min at 750 × *g*. Islets were then cultured overnight in RPMI 1640 medium containing 10% fetal bovine serum, 100 units/ml penicillin, and 100 μg/ml streptomycin (Invitrogen). The next morning, islets were incubated in EBSS containing 2.8 mmol/liter glucose at 37 °C for 30 min. Groups of three size-matched islets were then hand-picked and incubated in 0.3 ml of EBSS containing the indicated concentrations of glucose and ligands at 37 °C for 90 min. Following the static incubation experiments, supernatants were collected and stored at −20 °C until assayed for insulin using an electrochemiluminescence assay (Meso Scale Diagnostics, Rockville, MD). Data were calibrated to external standards and expressed as nanograms/ml insulin in the culture medium.

### Immunoprecipitation, Liquid Scintillation, and Denaturing PAGE Fluorography

HEK293 cells were adherently passaged to poly-d-lysine polymer-coated dishes. Transfection was performed using a mixture of FuGENE 6 reagent (Promega, Madison, WI) and pcDNA3.1 expression vector encoding the human GLP-1R-3×FLAG fusion protein. Following 48 h, cells were washed with 1× PBS and allowed to recover in supplemented Gibco 31053 media containing 0.5% fetal bovine serum prior to a 2-h treatment of 500 nm [^3^H]BETP at 37 °C. Cells were scraped from the dish to pellet material and to wash away unbound radioligand prior to immunoprecipitation. Cells were disrupted with sonication and removed from insoluble debris via centrifugation in lysis buffer (50 mm Tris-HCl pH 7.5, 150 mm NaCl, 1 mm EDTA pH 8.0, 1% Triton X-100, 1× Complete protease inhibitor (Roche Applied Science)), and 2 mm reduced l-GSH. All lysates were pre-cleared of immunoglobulin (IgG)-binding content for 2 h at 4 °C with mouse IgG-agarose gel (Sigma) prior to affinity purification using anti-FLAG M2-agarose gel (Sigma) and overnight rotation at 4 °C. The bead resins were washed three times following immunoprecipitation with lysis buffer and gravity filtration using empty Bio-Spin chromatography columns (Bio-Rad). Captured proteins were eluted from the M2 affinity gel or IgG control gel using SDS sample buffer at low speed centrifugation.

For liquid scintillation, immunopurified protein was added directly to Ultima Gold scintillator mixture (PerkinElmer Life Sciences), and radioactive decay was quantified with a Beckman LS3801 scintillation counter. Standard samples containing known amounts of radioactivity were also quantified to calculate the instrument counting efficiency for conversion to disintegrations/min. For fluorography, immunopurified protein was reduced with 2-mercaptoethanol at room temperature for electrophoretic separation. The 10% polyacrylamide gel containing radiolabeled samples was fixed with acetic acid in 2-propanol, agitated in Amersham Biosciences Amplify fluorographic agent (GE Healthcare), dried under heated vacuum, and exposed to x-ray MP Hyperfilm (PerkinElmer Life Sciences) at −80 °C. The film was developed using an automatic processor following 5–20 days of exposure.

### GLP-1R HEK293 Cell cAMP Assays

HEK293 cells expressing either wild-type human GLP-1R (NCBI accession number NP_002053) (20) or mutant C347A human GLP-1R-1×FLAG were used for measurement of cAMP accumulation. Cells were seeded into 96-well half-area black microplates in Gibco 31053 Dulbecco's modified Eagle's medium (Life Technologies, Inc.) containing 0.1% bovine serum albumin fraction. Peptide and compound treatments were diluted into medium containing isobutylmethylxanthine and added to cells for 30 min at 37 °C or 1 h at room temperature with 5% CO_2_. Cells were then assayed for cAMP accumulation using homogeneous time-resolved fluorescence technology (Cisbio Bioassays, Bedford, MA). Data were analyzed by the ratio method, calibrated to external standards, and reported as concentration of cAMP or expressed as percent cAMP compared with GLP-1(7–36)-NH_2_. To enable testing of small molecule compounds in higher throughput format, for some experiments, minor modifications to this protocol were made. Specifically, compound and GLP-1(9–36)-NH_2_ were dispensed acoustically (Echo, Labcyte, Sunnyvale, CA) into a total volume of 20 μl in Corning 384-well plates. GLP-1(9–36)-NH_2_ shift assays were performed with a concentration-response curve of GLP-1(9–36)-NH_2_ and a fixed dose of small molecules. Data were fit to the four-parameter logistic model (36), and ratios of potencies were used to quantify the fold-shift of GLP-1(9–36)-NH_2_ potency induced by allosteric modulators. Similarly, a concentration-response curve was generated for compounds in the presence of an EC_20_ of GLP-1(9–36)-NH_2_, and relative EC_50_ values were generated using the four-parameter model. Data were normalized using MIN and MAX controls, as described (36). Data analysis was performed using Screener (Genedata, San Francisco).

### Fluorescein-Maleimide Competition Assay

A competitive fluorescence-based assay for assessing the reactivity of compounds was developed and performed as described previously (37) with minor modifications. Briefly, a test compound was added to PBS (calcium/magnesium-free; Hyclone), followed by rapid addition of 100 nm GSH (ThermoFisher, Grand Island, NY), and then 50 nm fluorescein-5-maleimide (AnaSpec, San Jose, CA). The final DMSO concentration in the assay was 3.3% (v/v). Fluorescein emission was measured at 25 °C using a SpectraMax (Molecular Devices, Sunnyvale, CA). Test compounds were run in a concentration-response format, and equilibrium fluorescence was quantified. To obtain compound inhibitor constants for the reaction with GSH (*K*_GSH_), data were fit using PRISM (GraphPad, La Jolla, CA) to Equation 1,

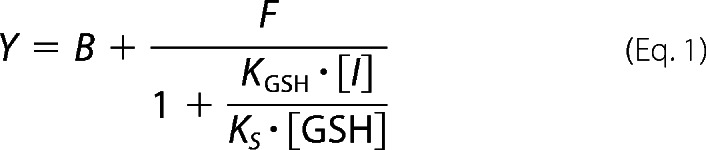
 where [*I*] is concentration of test compound; *K_S_* is the second order rate constant for reaction of fluorescein maleimide with GSH; *B* is basal fluorescence, and *F* is maximum fluorescence observed. *K_S_* was determined experimentally (16,000 m^−1^ s^−1^) and is concordant with published literature (37). Data were calculated as the harmonic means and standard deviations from three or more experiments.

### GSH Conjugation Followed by LC/MS

#### 

##### Reagents

Water and acetonitrile (ACN) were HPLC grade from Lab Scand (Dublin, Ireland). Formic acid (FA), ammonium hydrogen carbonate, phosphoric acid, sodium hydroxide, and l-GSH were from Sigma (Steinheim, Germany). For the sample preparation, a 100 mm sodium phosphate buffer, pH 7.4 (stored at 4 °C), 5 mm GSH in water, and stocks of the different target compounds at 5 mm in ACN were prepared.

##### Sample Preparation

Test and control samples were prepared and analyzed in parallel. Test samples contained 0.4 mm target compound and 1.4 mm GSH in phosphate buffer/ACN 70:30 in a total volume of 1.4 ml. Control samples contained 0.4 mm target compound in phosphate buffer/ACN 70:/30. Both solutions were incubated at 37 °C for 120 min in the HPLC autosampler and were analyzed at different times (standard times: 0, 30, 60, and 120 min).

### LC/MS Methods

An Agilent 1200 series rapid resolution LC/MSD SL system equipped with a solvent degasser, binary pump, auto sampler, column compartment, and a diode array detector (Agilent Technologies, Waldbronn, Germany) with XTerra MS column (50 × 2.1 mm, 3.5 μm; Waters Corp.) was used at 60 °C. The method involved gradient elution with water, 0.1% FA (solvent A) and ACN, 0.1% FA (solvent B) at a flow rate of 1.6 ml/min prior to the mass spectrometer, which was split at a ratio of 4:1. The A/B ratio was set at 80:20 as the initial elution condition, changing linearly to 0:100 in 0.8 min, and maintained at 0:100 from 0.8 to 1.0 min. Peaks were detected by absorbance at 214 nm and bandwidth at 16 nm, using the MS detector for identification. Electrospray mass spectrometry measurements were performed on a mass spectrometry detector quadrupole mass spectrometer (Agilent Technologies, Palo Alto, CA) interfaced to the HP1200 system, acquired simultaneously in positive and negative ionization modes (fragmentor 120 V, threshold spectral abundance 150, MS peak width 0.09 min) over the mass range of 100–800. Data acquisition and integration for LC-UV and MS detection were collected using ChemStation software (Agilent Technologies).

Injections were made at predetermined time points, which were then used to plot the consumption of target compound as a function of time. The MS method allowed for the simultaneous monitoring of conjugate formation.

### Cathepsin L Assay

Enzyme activity of the cysteine-protease cathepsin L (CatL) was quantified using human CatL (Calbiochem) and the fluorogenic substrate Z-Phe-Arg-7-amido-4-methylcoumarin (Z-Phe-Arg-AMC, Calbiochem). The assay buffer was 50 mm sodium phosphate buffer, pH 6.5, 2.5 mm EDTA, and 0.002 (w/v) Tween 20. Enzyme activity was measured at 25 °C in 384-well plates (Corning) using a Synergy (BioTek) plate reader with 360 nm excitation and 485 nm emission wavelengths. 20 μl of compound dilution series were incubated with 20 μl of CatL enzyme for 30 min. The assay was initiated by the addition of 20 μl of Z-Phe-Arg-AMC. Data were quantified after 10 min of reaction, and IC_50_ values were calculated (36). Final assay conditions were 2.6 nm CatL with 200 nm Z-Phe-Arg-AMC in 0.33% (v/v) DMSO. The assay was pharmacologically validated using the covalent CatL inhibitor (2*S*,3*S*)-*N*2-[(1*S*)-1-benzyl-2-(benzylamino)-2-oxo-ethyl]-*N*3-[2-(4-hydroxyphenyl)ethyl]oxirane-2,3-dicarboxamide (CAA0225; EMD Millipore) (38).

## Results

### 

#### 

##### BETP Potentiates cAMP Signaling and Stimulates Insulin Secretion in a Glucose- and GLP-1R-dependent Manner

**FIGURE 1.**
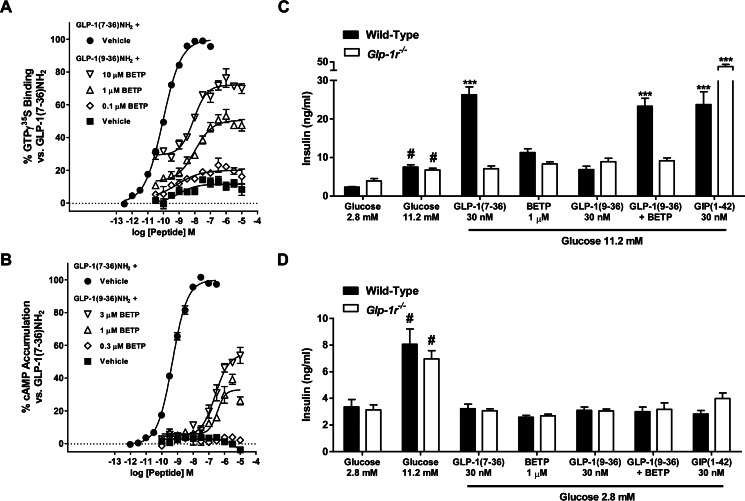
**BETP potentiates GLP-1(9–36)NH_2_-mediated cAMP signaling and stimulates insulin secretion in a glucose- and GLP-1R-dependent manner.** *A,* in the presence of GLP-1(9–36)-NH_2_, BETP enhances activation of Gα_s_ in GLP-1R-expressing membranes. Data show responses of varying concentrations of GLP-1(9–36)-NH_2_ alone (■) or in combination with 10 μm (▿), 1 μm (▵), or 0.1 μm (♢) of BETP. ● = GLP-1(7–36)-NH_2_. *B*, BETP potentiates GLP-1(9–36)-NH_2_-mediated cAMP accumulation in INS1 832-3 insulinoma cells. Intracellular concentrations of cAMP in response to varying concentrations of GLP-1(9–36)-NH_2_ alone (■) or in combination with 3 μm (▿), 1 μm (▵), or 0.3 μm (♢) of BETP are depicted. ● = GLP-1(7–36)-NH_2_. *C,* in cultures of isolated islets from wild-type (*black bar*) or *Glp-1r* KO (*white bar*) mice, BETP stimulates insulin secretion via a mechanism that requires high glucose (11.2 mm), GLP-1(9–36)-NH_2_, and the presence of the GLP-1R. Insulin levels in the media were measured following incubation of islets with the indicated concentrations of GLP-1(7–36)-NH_2_, BETP, GLP-1(9–36)-NH_2_, BETP plus GLP-1(9–36)-NH_2_, or glucose-dependent insulinotropic polypeptide (*GIP*)(1–42). *D*, insulin secretion is not enhanced by BETP in low glucose concentrations (2.8 mm). For each treatment, mean insulin concentrations were determined by measuring insulin in media from six independent wells containing three islets per well. ***, *p* < 0.001 using one-way ANOVA followed by Dunnett's comparison *versus* the respective 11.2 mm glucose group in *C*. For 11.2 mm glucose, #, *p* < 0.001 compared with the respective 2.8 mm glucose treatment in *C* and *D*. Data presented are representative of three to six independent experiments.

Studies were undertaken to determine the mechanism whereby BETP (19, 22) allosterically modulates the GLP-1R to enhance insulin secretion. To develop a proximal measure of receptor activation, a GLP-1R-containing cell membrane GTPγS binding assay was utilized. Here, upon receptor activation, the non-hydrolyzable guanine nucleotide analogue, [^35^S]GTPγS, binds Gα_s_. Accumulated radiolabeled Gα_s_ was then captured using anti-Gα_s_ antisera and measured. Consistent with the established ability of GLP-1R agonists to enhance insulin secretion by stimulating Gα_s_/adenylyl cyclase/cAMP signaling, BETP induced GTP-Gα_s_ binding in a cooperative manner with the inactive metabolite, GLP-1(9–36)-NH_2_ (Fig. 1*A*). Similarly, in INS1 832-3 insulinoma cells, BETP enhanced GLP-1(9–36)-NH_2_-induced cAMP accumulation in a concentration-dependent manner (Fig. 1*B*). The results showing BETP enhances GTP binding in the GLP-1R membrane assay and potentiates cAMP signaling in the pancreatic beta cell line extend the original findings from studies using GLP-1R-expressing HEK293 cells that showed BETP potentiates GLP-1(9–36)-NH_2_ but not GLP-1(7–36)-NH_2_ activity (22).

To investigate the insulin secretory mechanism of BETP, *ex vivo* cultures of isolated mouse islets were used. As BETP is a reactive molecule that has been shown to covalently modify several proteins (26), studies were undertaken to assess the specificity and glucose dependence of BETP in potentiating insulin secretion. Similar to previous results from rat islets incubated in high glucose concentrations showing BETP potentiated GLP-1(9–36)-NH_2_-stimulated insulin secretion (22), the combination of BETP and GLP-1(9–36)-NH_2_ increased insulin levels in static cultures of islets from wild-type mice (Fig. 1*C*). Importantly, the insulinotropic response did not occur in the presence of low glucose (Fig. 1*D*), results that are consistent with the established glucose dependence of GLP-1R activation to enhance insulin secretion. Furthermore, the co-treatment of BETP and GLP-1(9–36)-NH_2_ did not enhance insulin secretion in either low or high glucose concentrations in islets isolated from *Glp-1r* null mice (Fig. 1, *C* and *D*). As a control, the insulin secretory capacity of GLP-1R-deficient islets was confirmed by demonstrating that glucose-dependent insulinotropic polypeptide enhanced insulin secretion in these cultures (Fig. 1*C*). Together, the islet studies performed here indicate that the mechanism whereby BETP potentiates insulin secretion requires the presence of high glucose, the GLP-1R, and a peptide ligand.

##### Electrophilic Mechanism of Action of BETP

**FIGURE 2.**
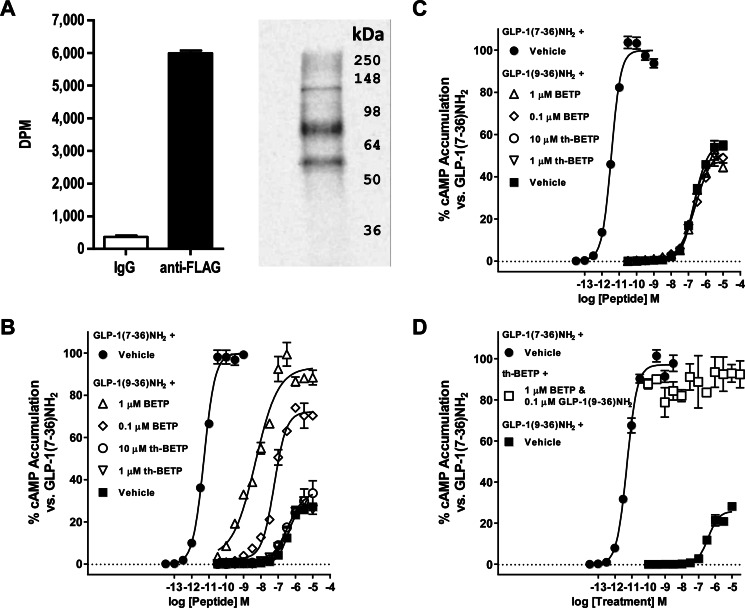
**GLP-1R binding and activation by BETP requires its electrophilic nature.** *A,* affinity purification of GLP-1R-3XFLAG from HEK293 cells treated with [^3^H]BETP shows labeling of the receptor. Liquid scintillation disintegrations/min from immunoprecipitation assays using IgG control (*white bar*) or anti-FLAG antibody (*black bar*)-conjugated beads are shown. Under reducing conditions, 10% PAGE fluorography of anti-FLAG immunopurified proteins shows a banding pattern consistent with migration of the GLP-1R. *B,* in HEK293 cells expressing the GLP-1R, the non-reactive analogue of BETP, th-BETP, does not potentiate GLP-1(9–36)-NH_2_-stimulated cAMP accumulation. Data show results from assays testing varying concentrations of GLP-1(9–36)-NH_2_ alone (■) or in combination with 1 μm (▵) or 0.1 μm (♢) BETP or with 10 μm (○) or 1 μm (▿) th-BETP. ● = GLP-1(7–36)-NH_2_. *C*, BETP does not potentiate GLP-1(9–36)-NH_2_ activity in HEK293 cells expressing the mutant C347A GLP-1R-1XFLAG. Accumulation of intracellular cAMP was stimulated by varying concentrations of GLP-1(9–36)-NH_2_ alone (■) or in combination with 1 μm (▵) or 0.1 μm (♢) BETP or with 10 μm (○) or 1 μm (▿) th-BETP. ● = GLP-1(7–36)-NH_2_. *D,* in an antagonist assay format, GLP-1R activation in HEK293 cells by BETP is not blocked by co-incubation of the non-reactive analogue th-BETP. Varying concentrations of the non-reactive th-BETP analogue (□) do not blunt intracellular cAMP accumulation induced by the combination of BETP (1 μm) and GLP-1(9–36)-NH_2_ (0.1 μm). ● = GLP-1(7–36)-NH_2_, and ■ = GLP-1(9–36)-NH_2_. All data are fit to the four-parameter logistic equation, and the EC_50_ value for BETP in cAMP accumulation assays using GLP-1R-expressing HEK293 cells is presented in Table 1.

Because of the inherent reactivity of BETP (16), studies were performed to assess whether BETP irreversibly modifies the GLP-1R. For these experiments, a radiolabeled analogue of BETP was synthesized ([^3^H]BETP) and incubated with HEK293 cells expressing human GLP-1R that contains a carboxyl-terminal FLAG epitope (35). Anti-FLAG affinity purification of lysates from cells treated with [^3^H]BETP showed high disintegrations/min from GLP-1R-FLAG-expressing cells *versus* purifications using mouse immunoglobulin control beads (Fig. 2*A*). Furthermore, PAGE fluorography of lysates under denaturing conditions demonstrated bands consistent with the expected migration pattern of glycosylated GLP-1R (Fig. 2*A*) (35). The finding that [^3^H]BETP irreversibly labels the GLP-1R is in concordance with studies showing BETP covalently binds Cys-347 (26).

**FIGURE 3.**
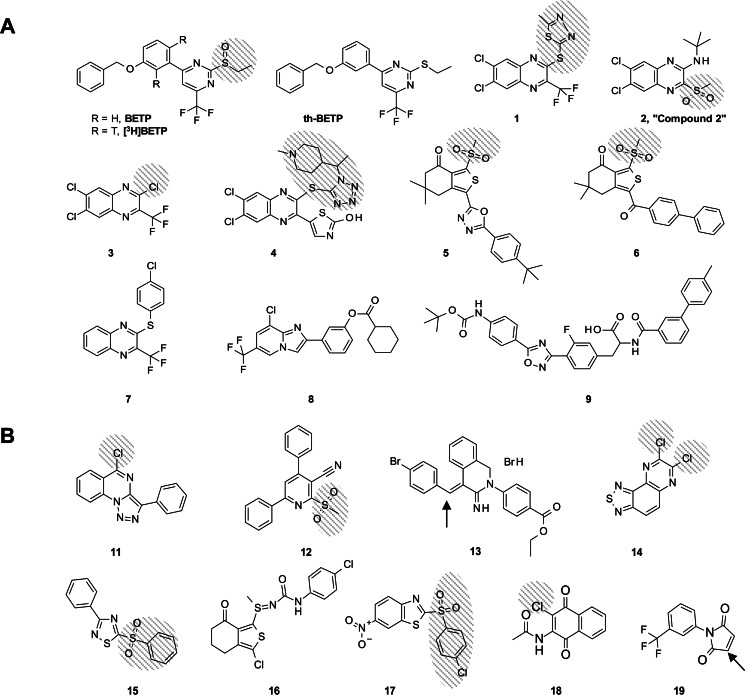
*A,* chemical structures of known GLP-1R-activating chemotypes are depicted. The leaving groups from nucleophilic attack are *shaded*. EC_50_ values for each molecule in cAMP accumulation assays using GLP-1R-expressing HEK293 cells are presented in Table 1. *B,* chemical structures of the electrophilic compounds evaluated for the ability to modulate GLP-1R activity are presented. The leaving groups from nucleophilic attack are *shaded* and nucleophilic attack to double bonds is represented with an *arrow*. EC_50_ values for each molecule are presented in Table 2.

To evaluate whether covalent interaction of BETP with the GLP-1R is required for allosteric modulation of receptor activity, a non-reactive structurally similar analogue of BETP that contains thioether in place of the sulfoxide moiety was synthesized (th-BETP, Fig. 3*A*). For these experiments, HEK293 cells overexpressing the human GLP-1R were used, and as opposed to studies of isolated islets or insulinoma cell lines, GLP-1(9–36)-NH_2_ shows partial stimulation of cAMP accumulation (21–23). Co-treatment of th-BETP and GLP-1(9–36)-NH_2_ to these cells showed no additional increase in cAMP accumulation compared with peptide alone (Fig. 2*B* and Table 1). Furthermore, these assays confirmed the previously reported discovery that Cys-347 of the GLP-1R is required for BETP function, as cells expressing the C347A mutant were not potentiated by BETP (Fig. 2*C* and Table 1). The concentration-response curves shown for the mutant assay were calculated as a percentage of maximum stimulation by GLP-1(7–36)-NH_2_; the apparent increase in *E*_max_ of GLP-1(9–36)-NH_2_ may therefore reflect subtle differences in the efficacy of the cognate ligand in wild-type *versus* C347A GLP-1R cells, a phenomenon that was also previously observed (26).

**TABLE 1:** **Reactivity and pharmacological characterization of known small molecule GLP-1R activators** SR_1_ = 5-methyl-1,3,4-thiadiazole-2-thiol; SR_2_ = 1-[1-(1-methyl-4-piperidyl)ethyl]tetrazole-5-thiol. Compound EC_50_ values are compound potencies determined from cAMP accumulation assays using wild-type (WT) or C347A GLP-1R-1×FLAG expressing HEK293 cells in the presence of 220 nm GLP-1(9–36)-NH_2_. 10-Point compound concentration-response curves were run from a maximum of 30 μm, with 3-fold dilutions. Data were normalized using min (220 nm GLP-1(9–36)-NH_2_ + DMSO = 0%) and max (220 nm GLP-1(9–36)-NH_2_ + 1 μm reference compound = 100%) controls (36). Potency values are represented as geometric means, standard deviations (S.D.), and number of independent replicates (*n*). Compound efficacy values (*E*_max_) are represented as arithmetic means, standard deviations (S.D.), and number of independent replicates (*n*). Representative curves are presented in Fig. 5, *A* and *C*. NA = not applicable. GLP-1(9–36)-NH_2_ fold shift values are calculated as the ratio of GLP-1(9–36)-NH_2_ potencies in the presence of 10 μm test compound *versus* DMSO vehicle. 10-Point GLP-1(9–36)NH_2_ compound concentration-response curves were run from a maximum of 75 μm, with 3-fold dilutions. Data were normalized using min (DMSO = 0%) and max (75 μm GLP-1(9–36)-NH_2_ + 1 μm reference compound = 100%) controls (36). Potency values are represented as geometric means, standard deviations (S.D.), and number of independent replicates (*n*). GLP-1(9–36)-NH_2_ efficacy values (*E*_max_) are represented as arithmetic means, standard deviations (S.D.), and number of independent replicates (*n*).

Compound	MS of GSH adduct, (*M*_r_)	Functional group displaced by GSH	Compound, EC_50_ WT (nm) (S.D., *n*)	Compound, *E*_max_% (S.D., *n*)	GLP-1(9–36), fold shift, WT (S.D., *n*)	GLP-1(9–36), *E*_max_, % (S.D., *n*)	EC_50_, C347A (nm) (S.D., *n*)	Compound, *E*_max_, % (S.D., *n*)	GLP-1(9–36), fold shift, C347A (S.D., *n*)	GLP-1(9–36), *E*_max_, % (S.D., *n*)	Refs.
BETP	635	SOEt	1300 (700, 8)	74 (8, 10)	75 (60, 146)	110 (30, 146)	>30000 (NA, 9)	NA	1.1 (0.2, 10)	12 (4, 10)	19, 22
th-BETP			>30000 (NA, 4)	NA	<1 (NA, 3)	4.3 (1, NA)	>30000 (NA, 3)	NA	1.3 (0.4, 3)	11 (1, 3)	25
1	573	SR_1_	860 (300, 4)	80 (10, 4)	150 (100, 124)	110 (30, 124)	>30000 (NA, 4)	NA	1.7 (0.4, 4)	30 (2, 4)	17, 18
**2**	574	SO_2_Me	25 (5, 3)	100 (10, 3)	650 (700, 5)	73 (20, 5)	>30000 (NA, 3)	NA	2.8 (0.05, 3)	11 (3, 3)	17, 18
**3**	573*^a^*	Cl	4700 (1000,4)	58 (10, 4)	15 (3, 4)	70 (10, 4)	>30000 (NA, 4)	NA	0.97 (0.2, 4)	13 (3, 4)	18
**4**	602	SR_2_	98 (30, 3)	85 (8, 3)	150 (80, 4)	54 (10, 4)	>30000 (NA, 4)	NA	2.1 (2, 4)	2.9 (2, 4)	27
**5**	685	SO_2_Me	1500 (1000, 4)	66 (21, 4)	20 (5, 4)	90 (8, 4)	>30000 (NA, 4)	NA	1.6 (0.4, 4)	16 (1, 4)	28
**6**	665	SO_2_Me	1430 (NA, 1)	17 (NA, 1)	14 (NA, 1)	36 (NA, 1)	>30000 (NA, 1)	NA	2.6 (NA, 1)	14 (NA, 1)	28
**7**			>30000 (NA, 4)	NA	NA*^b^* (NA, 4)	NA*^b^* (NA, 4)	>30000 (NA, 4)	NA	1.7 (0.3, 4)	14 (5, 4)	29
**8**			>30000 (NA, 4)	NA	<1 (NA, 3)	NA (NA, 3)	>30000 (NA, 3)	NA	1.4 (0.5, 3)	8.8 (1, 3)	30
**9**			1500 (1000, 4)	93 (50, 4)	57 (20, 7)	110 (30, 7)	1400 (500, 4)	93 (10, 4)	130 (30, 4)	106 (12, 4)	36

*^a^* Parent compound was still detected after 60 min.

*^b^* Data could not be calculated as curves were not obtained in the shift assay.

Studies designed to assess the ability of the non-reactive th-BETP analogue to antagonize BETP showed no inhibition of BETP. Here, near maximal stimulation of cAMP production in wild-type GLP-1R HEK293 cells via combination of BETP and GLP-1(9–36)-NH_2_ was not blunted by co-addition of increasing concentrations of th-BETP (Fig. 2*D*). The lack of activity of the non-reactive th-BETP analogue in potentiator assays and the inability of this molecule to blunt BETP action support the assertion that there is not a conventional small molecule binding pocket in the local proximity of Cys-347 of the GLP-1R.

##### Known Small Molecule GLP-1R Activators Are Electrophilic

To determine whether other modulators of the GLP-1R activate the receptor by a covalent mechanism, several previously reported compounds that contain potentially electrophilic positions (compounds **1**-**8**, Fig. 3*A*) were selected. Compound **9** and th-BETP were used as negative controls because these molecules are not predicted to be reactive. Because the reactivity of BETP to nucleophilic cysteine of GSH has been shown previously (25), a GSH-based conjugation assay is likely an appropriate surrogate for evaluating the potential reactivity of compounds toward free cysteines of the GLP-1R. Thus, BETP, th-BETP, and compounds **1–9** were incubated with GSH (39), and the progression of reactions over time (disappearance of parent compound and appearance of the corresponding adduct formed with GSH) was measured by LC/MS. Under the conditions used, BETP disappeared within 60 min (Table 1). It must be noted that the *t*_½_ of BETP conjugation to GSH was previously determined as <0.5 min (25), corresponding to a second order rate constant of >0.5 m^−1^ s^−1^. Results for the other molecules show compounds **1–6** are also reactive to cysteine of GSH; interestingly, each parent compound disappeared within 10 min (data not shown) except for compound **3**. Compounds **7** and **8** remained unaltered after 2 h and thus are deemed non-reactive. As expected, compound **9** and th-BETP were also chemically stable. The results showing compound **4** was as unstable in these experiments as quinoxalines **1**-**3** is of interest because compound **4** has been reported to be orally efficacious (27, 40), although its apparent electrophilicity suggests poor *in vivo* pharmacokinetic properties, as has been shown for BETP (25). It should be pointed out that the GSH conjugation assay was performed as described previously (39), using buffer containing organic solvent, conditions that are not representative of *in vivo* physiology. However, it is indicative of the intrinsic reactivity of the compounds to any cysteine within proximity and therefore predictive of short plasma half-lives when conjugation is observed in this assay; results from the *in vitro* assays performed here confirm the GSH conjugation mechanism for BETP and also indicate that several known modulators of the GLP-1R are as electrophilic as BETP.

An advantage of the GSH conjugation assay is that it utilizes LC/MS, which enabled us to determine the position of the nucleophilic attack, the structure of the leaving group (see *shaded atoms* in Fig. 3*A*), and the structure of the new species formed for each reactive compound from the mass of the new adduct (Table 1). Similar to BETP, the adduct formed by incubation of compound **2** with GSH results from addition of GSH to the quinoxaline ring and elimination of the sulfonyl group. This structure is consistent with the mass obtained by LC/MS. A similar mechanism of addition-elimination to electron-poor aromatic rings is presumed for compounds **1** and **3–6**, with elimination of a thiol when GSH reacts with compounds **1** and **4**, elimination of a sulfone when it reacts with compounds **5** and **6**, and elimination of chloride for compound **3**.

To determine whether these electrophilic GLP-1R modulators activate the receptor via a mechanism similar to BETP, the compounds were tested in cAMP accumulation assays using cells expressing either wild-type or the C347A GLP-1R. Results of these studies are presented in Fig. 5, *A* and *C,* and Table 1. We determined the capacity of these molecules to potentiate GLP-1(9–36)-NH_2_-mediated cAMP accumulation in GLP-1R-expressing cells using two distinct assays. The first assay (Fig. 5 and referred to as compound EC_50_ in Tables 1 and 2) measures compound potency in the presence of an EC_20_ amount of GLP-1(9–36)-NH_2_. Any observed potency and efficacy enhancements in this assay reflect α and β factor modulation of orthosteric and allosteric ligands (41). The second assay (referred to as GLP-1(9–36)-NH_2_ shift in Tables 1 and 2) measures the EC_50_ and maximal efficacy of GLP-1(9–36)-NH_2_ in combination with a fixed 10 μm concentration of compound. This assay provides an estimate of the maximal β-induced increases in efficacy and α-induced enhancements in affinity of the allosteric modulators for GLP-1(9–36)-NH_2_ (41). Analogous to BETP, compounds **1–6** potentiated GLP-1(9–36)-NH_2_ activity on the wild-type receptor but not in cells expressing the mutant GLP-1R that lacks Cys-347. Together with findings from the GSH conjugation studies, the functional data support the required role of this cysteine in the allosteric mechanism used by the various chemotypes. It is important to note that the mutant receptor was functional as compound **9**, a non-electrophilic molecule reported to activate the GLP-1R (42), potentiated GLP-1(9–36)-NH_2_ in both wild-type and C347A assays (Fig. 5, *A* and *C,* and Table 1). To our knowledge, this is the first study showing potentiation of GLP-1(9–36)-NH_2_ by a compound from this series. Furthermore, these results are significant because the data indicate potentiation of the GLP-1R can be achieved without covalent modification.

**TABLE 2:** **Reactivity and pharmacological characterization of electrophilic compounds that activate GLP-1R in a cysteine 347-dependent manner** Compound EC_50_ values are compound potencies determined from cAMP accumulation assays using wild-type (WT) or C347A GLP-1R-1×FLAG expressing HEK293 cells in the presence of 220 nm GLP-1(9–36)-NH_2_. 10-Point compound concentration-response curves were run from a maximum of 30 μm, with 3-fold dilutions. Data were normalized using min (220 nm GLP-1(9–36)-NH_2_ + DMSO = 0%) and max (220 nm GLP-1(9–36)-NH_2_ + 1 μm reference compound = 100%) controls (36). Potency values are represented as geometric means, standard deviations (S.D.), and number of independent replicates (*n*). Compound efficacy values (*E*_max_) are represented as arithmetic means, standard deviations (S.D.), and number of independent replicates (*n*). Representative curves are presented in Fig. 5, *B* and *D*. NA = not applicable. GLP-1(9–36)-NH_2_ fold shift values are calculated as the ratio of GLP-1(9–36)-NH_2_ potencies in the presence of 10 μm test compound *versus* DMSO vehicle. 10-Point GLP-1(9–36)-NH_2_ compound concentration-response curves were run from a maximum of 75 μm, with 3-fold dilutions. Data were normalized using min (DMSO = 0%) and max (75 μm of GLP-1(9–36)-NH_2_ + 1 μm of reference compound = 100%) controls (36). Potency values are represented as geometric means, standard deviations (S.D.), and number of independent replicates (*n*). GLP-1(9–36)-NH_2_ efficacy values (*E*_max_) are represented as arithmetic means, standard deviations (S.D.), and number of independent replicates (*n*).

Compound	MS of GSH adduct (*M*_r_)	Functional group displaced by GSH	Compound, EC_50_, WT (nm) (S.D., *n*)	Compound, *E*_max_, % (S.D., *n*)	GLP-1(9–36), fold shift WT (S.D., *n*)	GLP-1(9–36), *E*_max_, % (S.D., *n*)	Compound, EC_50_, C347A (nm) (S.D., *n*)	Compound, *E*_max_, %(S.D., *n*)	GLP-1(9–36), fold shift C347A (S.D., *n*)	GLP-1(9–36), *E*_max_, % (S.D., *n*)
**11**	551*^a^*	Cl	7000 (900, 4)	26 (5, 4)	6.7 (1, 4)	53 (10, 4)	>30000 (NA, 4)	NA	1.5 (0.5, 4)	16 (5, 4)
**12**	561*^a^*	SO_2_Me	6000 (700, 4)	11 (1, 4)	4.0 (0.6, 4)	35 (2, 4)	>30000 (NA, 4)	NA	1.3 (0.04, 4)	13 (3, 4)
**13**	768	None*^b^*	1000 (300, 3)	21 (10, 4)	38 (20, 3)	23 (10, 3)	>30000 (NA, 4)	NA	8.0 (5, 4)	9 (4, 4)
**14**	527	Cl	>30,000*^c^* (NA, 4)	NA	4.2 (0.6, 4)	45 (10, 4)	>30000 (NA, 4)	NA	1.5 (0.3, 4)	18 (5, 4)
**15**	465	SO_2_Ph	1300 (NA, 2)	44 (NA, 2)	64 (10, 4)	54 (11, 4)	>30000 (NA, 4)	NA	5.1 (0.9, 4)	20 (7, 4)
**16**			5200 (1000, 4)	58 (5, 4)	13 (2, 4)	81 (6, 4)	>30000 (NA, 4)	NA	2.1, (0.5, 4)	29 (6, 4)
**17**	485	SO_2_Ph-p-Cl	>30,000*^c^* (NA, 4)	NA	13 (5, 4)	24 (9, 4)	>30000 (NA, 4)	NA	1.7 (0.5, 4)	16 (3, 4)
**18**	520	Cl	>30,000 (NA, 2)	NA	0.85 (NA, 2)	3 (NA, 2)	>30000 (NA, 4)	NA	0.97 (NA, 2)	13 (NA, 2)
**19**	548	None*^b^*	>30,000 (NA, 2)	NA	0.88 (NA, 2)	2 (NA, 2)	>30000 (NA, 4)	NA	1.4 (NA, 2)	10 (NA, 2)

*^a^* Parent compound was still detected after 60 min.

*^b^* Addition of GSH to the double bond is shown.

*^c^* Compounds gave quantifiable activity in GLP-1(9–36)-NH_2_ shift assay but not in compound dose-response assay. Compounds demonstrated activity in WT (but not C347A) cells, but this was weakly potent and refractory to curve fitting.

##### Innate Reactivity Influences Compound-induced Potentiation of the GLP-1R

**FIGURE 4.**
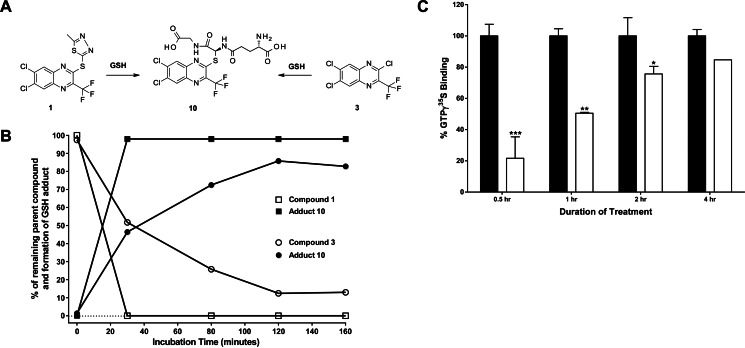
**Intrinsic chemical reactivity of electrophilic modulators of the GLP-1R influences receptor activation.** *A,* nucleophilic attack of GSH to quinoxalines **1** and **3** shows formation of the same adduct **10**. *B,* compounds **1** and **3** display different rates of reactivity in a GSH conjugation assay. Data show disappearance of **1** over time (□), formation of adduct **10** over time when **1** is incubated with GSH (■), disappearance of **3** over time (○), and formation of adduct **10** when **3** is incubated with GSH (●). *C*, time course studies show Gα_s_ activation of the GLP-1R by the quinoxalines. In combination with GLP-1(9–36)-NH_2_ (3 μm), compounds **1** (3 μm; *black bar*) and **3** (3 μm; *white bar*) enhance activation of Gα_s_ in GLP-1R-expressing membranes. Data show mean responses over time of the compounds tested in duplicate in combination with GLP-1(9–36)-NH_2_. ***, *p* < 0.0001 using one-way ANOVA followed by pairwise Student's *t* tests to respective durations of treatment. **, *p* < 0.01; *, *p* < 0.05. Data presented are representative of three independent experiments.

**FIGURE 5.**
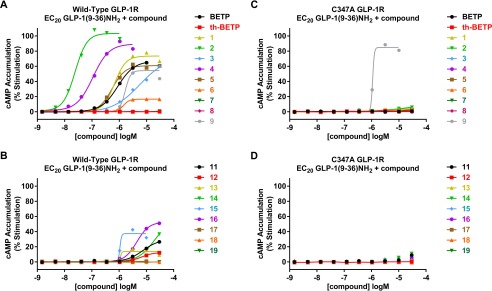
**Requirement of Cys-347 of the GLP-1R for positive allosteric modulation by small molecules.** GLP-1(9–36)-NH_2_-induced cAMP accumulation was measured in wild-type (*A* and *B*) and C347A (*C* and *D*) GLP-1R-expressing HEK293 cells in the presence of small molecule GLP-1R ligands. Cells were treated with serial dilutions of the compounds in the presence of an EC_20_ concentration of GLP-1(9–36)-NH_2_. Resultant cAMP was quantified and data were normalized and fit to the four-parameter logistic equation as described under “Experimental Procedures.” Data graphed exemplify single representative experiments. Summarized data and statistics from multiple experiments are presented in Tables 1 and 2.

Compounds **1** and **3** require Cys-347 of the GLP-1R for activity and are predicted to generate the same adduct upon reacting with a cysteine and therefore to have similar potentiation activity (Table 1 and Fig. 4*A*). However, these molecules showed different modulator activities in the wild-type GLP-1R assay (Fig. 5*A* and Table 1). The different electrophilic character of these compounds appears to account for the difference. In the GSH conjugation assay, adduct **10** was obtained from both compounds; however, the parent species was still detected after 60 min of incubation of GSH with compound **3** but not for compound **1** (parent had disappeared after 30 min, first time point of this experiment) (Fig. 4*B*), suggesting the rate of adduct formation is slower for compound **3**. To confirm these results using conditions more similar to those of the GLP-1R cellular assay, the molecules were tested in an aqueous assay (phosphate buffered saline, pH 7.4) where compounds compete with fluorescein-5-maleimide for reaction with GSH (37). Consistent with the LC/MS findings, calculation of the rate constant for the reaction of compounds **1** and **3** with GSH (*K*_GSH_) demonstrated that compound **1** is more reactive (**1** = 13 m^−1^ s^−1^ (S.D. = 10, *n* = 11) than compound **3** = <1 m^−1^ s^−1^ (*n* = 4) (*p* = 0.0015 Mann-Whitney *U* test)). BETP (19 m^−1^ s^−1^ (S.D. = 10, *n* = 6)) was used as a positive control in these experiments. Taken together, these data indicate compound **3** may need a longer incubation time in the functional assays to allow more adduct formation. Thus, time course studies using the GLP-1R [^35^S]GTPγS binding assay were performed and demonstrate that prolonged incubation of compound **3** induced a similar response to compound **1** (Fig. 4*C*).

##### Discovery of New GLP-1R Allosteric Modulators

Because the covalent binders shown above represent three different chemotypes, and because the common feature shared by these molecules is the presence of an electrophile within each compound, several structurally distinct reactive compounds that had been previously identified in GLP-1R screening campaigns were tested for their ability to modulate GLP-1R activity in a Cys-347-dependent manner. These molecules were discovered as putative GLP-1R potentiators from screens of the Lilly chemical library using GLP-1R-expressing HEK293 cells where compounds were tested in the presence of a submaximal concentration of GLP-1(9–36)-NH_2_. The chemical structures of these compounds are depicted in Fig. 3*B*, and the potentiator results are presented in Fig. 5, *B* and *D,* and Table 2. A variety of compounds showed potentiation of GLP-1(9–36)-NH_2_ at the wild-type receptor. However, in all cases, compound activity was lost in assays testing the mutant GLP-1R. Consistent with the covalent mechanism, most compounds were confirmed to be reactive in the GSH conjugation assay (Table 2). As negative controls, compounds **18** and **19** were unstable in the presence of GSH and did not potentiate the activity of GLP-1(9–36)-NH_2_ in the wild-type GLP-1R assay. These are the most reactive compounds in our studies (**18** = 22,000 m^−1^ s^−1^ (S.D. = 9000, *n* = 5); **19** = 1200 m^−1^ s^−1^ (S.D. = 500, *n* = 6)), and it is not known whether the lack of PAM activity is due to failure of the molecules to covalently bind the GLP-1R or because the adduct formed is insufficient to facilitate structural changes in the receptor that allow its activation by GLP-1(9–36)-NH_2_. It is also possible that these compounds cannot access the intracellular location of Cys-347. Interestingly, as expected for a covalent binder, compound **16** showed activity in the Cys-347-containing wild-type receptor but not in the mutant; however, this molecule did not react with GSH in the conjugation assay or in the fluorescein-5-maleimide competition assay (**16** = <1 m^−1^ s^−1^ (*n* = 3)). Precipitation of the compound was observed in the conjugation assay followed by LC/MS. Although low solubility could explain the lack of reactivity of this compound with GSH, other hypotheses for this discrepancy can be envisioned as follows: 1) compound **16** activates the receptor by covalent modification, but Cys-347 in the local cellular environment is slightly more nucleophilic than GSH in the conjugation assays; 2) compound **16** is a pro-drug and requires cellular metabolism to produce the active species.

In addition to Cys-347 of the GLP-1R, Cys-438 was also shown to be covalently modified by BETP, but neither BETP nor compound **2** showed reduced abilities to potentiate GLP-1(9–36)-NH_2_ activity in C438A GLP-1R assays (26). Although it is clear that the newly identified reactive GLP-1R potentiators in our report require Cys-347 for activity, it is possible these electrophiles also bind Cys-438. Although the previous studies showed mutation of Cys-438 does not alter PAM activity, even for the ability of BETP and compound **2** to potentiate oxyntomodulin (26), it cannot be absolutely excluded that formation of an adduct at this position could positively or negatively influence Cys-347-required activity.

To assess the promiscuity of the new PAMs toward proteins containing nucleophilic cysteine residues, we investigated the inhibitory action of BETP and compounds **11–17** on the activity of several enzymes. These studies utilized several kinases known to be irreversibly inhibited by reactive compounds (EGFR (43), CDK7 (44), BTK (45), and ITK (45)) and the cysteine protease CatL. The results are summarized in Table 3. Weak inhibitory activity was observed for compounds **13** and **15–17**, although none of the compounds inhibited all of the enzymes. The kinases tested contain non-catalytic cysteine residues in their active sites. Thus, non-inhibitory covalent modification of cysteines by inactive compounds cannot be ruled out from these experiments. For CatL, the cysteine-thiol is fundamental for catalysis, and therefore, covalent modification of active site CatL will parallel enzyme inhibition. The lack of activity of BETP and compounds **11–14** against CatL shows that these electrophiles do not react with the catalytic cysteine of this enzyme, whereas the covalent inhibitor CAA0225 exhibited potent activity (IC_50_ = 6 nm (S.D. = 1, *n* = 4)).

**TABLE 3:** **Inhibition of several enzymes by electrophiles**

Compound	BTK,*^a^* IC_50_	ITK,*^a^* IC_50_	EGF receptor,*^a^* IC_50_	CDK7,*^b^* IC_50_	CatL,*^c^* IC_50_
	μ*m*	μ*m*	μ*m*	μ*m*	μ*m*
BETP	>100	>100	>100	>20	>33
**11**	>100	>100	>100	>20	>33
**12**	>100	>100	>100	>20	>33
**13**	>100	9.8	>100	11.6	>33
**14**	>100	>100	>100	>20	>33
**15**	>100	>100	>100	>20	25
**16**	23	>100	>100	>20	12
**17**	>100	>100	>100	>20	8

*^a^* Active site occupancy was quantified using TR-FRET (LanthaScreen, ThermoFisher) with compound preincubation of 180 min and a maximal compound concentration of 100 μm, essentially as described (64).

*^b^* Enzymatic activity was quantified using radiometric filter binding with a compound preincubation of 180 min and a maximal compound concentration of 20 μm, as described previously (65).

*^c^* Cathepsin L activity was quantified as described under “Experimental Procedures.” Data are the mean of three experiments.

**FIGURE 6.**
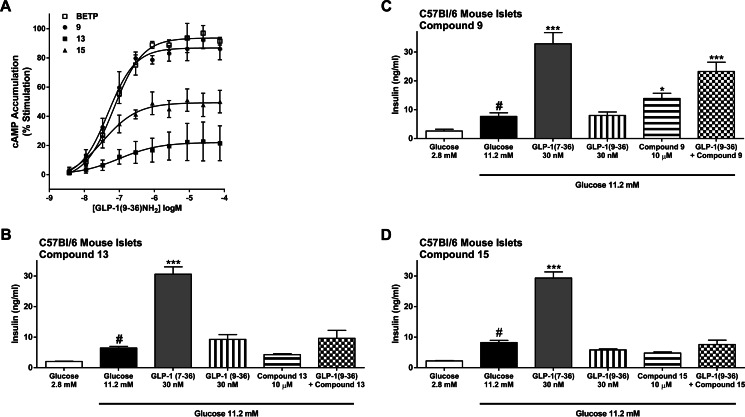
**Comparison of compound-stimulated cAMP generation in HEK293 cells expressing GLP-1R *versus* insulin secretion from isolated pancreatic islets.** *A*, GLP-1(9–36)-NH_2_-induced cAMP accumulation was measured in GLP-1R-expressing cells in the presence of small molecule GLP-1R ligands. Cells were treated with varying concentrations of GLP-1(9–36)-NH_2_ in the presence of 10 μm BETP (□), compound **9** (●), compound **13** (■), or compound **15** (▴), and cAMP was quantified. Data were normalized and fit to the four-parameter logistic equation as described under “Experimental Procedures.” Data were graphed as the mean (±S.D.) of three independent experiments. *B–D,* insulin levels in media from cultures of wild-type (C57/Bl6 mice) islets treated with GLP-1(7–36)-NH_2_, GLP-1(9–36)-NH_2_, compound **9**, **13**, or **15** alone, or GLP-1(9–36)-NH_2_ plus each compound. Glucose-stimulated insulin secretion is shown for each islet preparation (2.8 *versus* 11.2 mm concentrations of glucose), and all compounds were tested in the presence of high glucose. Data are from three islet experiments where mean insulin concentrations for each treatment group were determined by measuring insulin in media from six independent wells containing three islets per well. ***, *p* < 0.001, and *, *p* < 0.05 using one-way ANOVA followed by Dunnett's comparison compared with the respective 11.2 mm glucose treatment, and # = *p* < 0.001 compared with the respective 2.8 mm glucose treatment.

Based on potency in the GLP-1R-expressing cellular assays, compounds **13** and **15** were selected for further characterization. Compared with the potentiator activity of BETP and compound **9**, two chemotypes that have been structurally optimized using GLP-1R activity assays, compounds **13** and **15** showed lower efficacy in assays where a fixed concentration of compound (10 μm) was tested in the presence of various concentrations of GLP-1(9–36)-NH_2_ (Fig. 6*A*). Consistent with lower *E*_max_ values, these molecules also do not potentiate insulin secretion in cultures of isolated pancreatic islets from normal mice (Fig. 6, *B–D*). Although larger data sets are needed to define the level and kinetics of cAMP induction in GLP-1R HEK293 cells that correlate with the insulinotropic actions of GLP-1R PAMs, it is likely that a minimum threshold of cAMP accumulation is needed.

**FIGURE 7.**
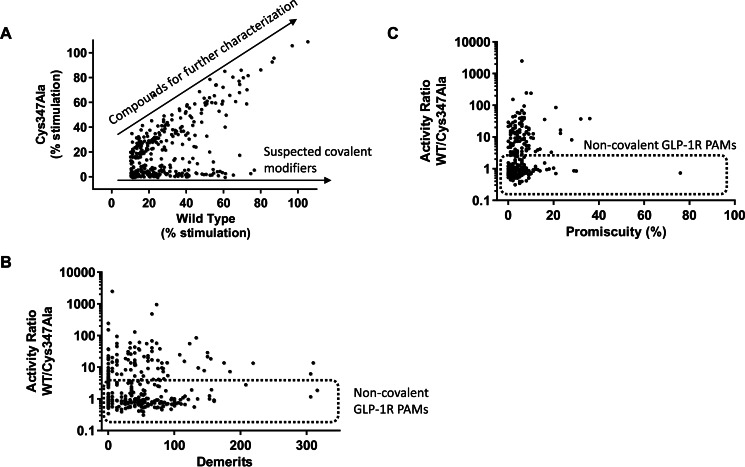
**Comparative analysis of GLP-1R Cys-347-dependent and Cys-347-independent non-peptide ligands.** *A*, 560 putative GLP-1R ligands were tested at 30 μm in the presence of an EC_20_ (220 nm) of GLP-1(9–36)-NH_2_ for the mobilization of intracellular cAMP in C347A and wild-type (*WT*) GLP-1R-expressing HEK293 cells. The 416 compounds with >10% stimulation in WT cells are graphed. Two clear populations of compounds are annotated on the scatter plot as follows: compounds with approximately equivalent WT and C347A GLP-1R PAM activity (compounds for further characterization), and compounds with high activity in WT GLP-1R and close to no activity in C347A GLP-1R assays (suspected covalent modifiers). *B*, activity ratios were calculated from *A* as % stimulation in WT cells divided by % stimulation in C347A cells. This activity ratio was plotted *versus* the calculated medchem demerits as described by Bruns and Watson (46). *Non-covalent GLP-1R PAMs box* annotates compounds with approximately equivalent activity in WT and C347A assays. *C*, WT/C347A activity ratios were plotted *versus* compound promiscuity. For a given compound, promiscuity was defined as the percentage of assay activities from total number of tested assays. Active was defined as either a concentration response value <10 μm and/or a single point test of >90% stimulation or inhibition. Compounds were not included in this analysis unless they had been tested in >20 assays. *Non-covalent GLP-1R PAMs box* annotates compounds with approximately equivalent activity in WT and C347A assays.

Following the discovery and characterization of the new reactive GLP-1R PAMs, we examined the general prevalence of Cys-347-preferring electrophiles among GLP-1R ligands by testing a large selection of compounds (identified from our screens) in the GLP-1R C347A assay. 560 molecules, identified as putative hits from the GLP-1(9–36)-NH_2_ potentiation screen, were tested at 30 μm in the presence of an EC_20_ concentration of GLP-1(9–36)-NH_2_ in both wild-type and C347A GLP-1R-expressing HEK293 cells. 416 compounds showed >10% stimulation in wild-type GLP-1R cells, and this was used as a threshold for subsequent analyses. Correlation of these 416 screening hits in wild-type and C347A GLP-1R cells indicates two clear populations of compounds: a population with equivalent activity in wild-type and C347A cells (∼55% of compounds) and those with activity in wild-type GLP-1R cells but minimal activity in C347A cells (Fig. 7*A*). Further assessment of these data was performed by correlating the ratio of activities (wild-type/C347A) with “medchem demerits”. Medchem demerits derive from a rules-based computational tool for identifying potentially reactive and promiscuous molecules based on substructure searching (46). Compounds with >100 demerits are considered undesirable. Our analysis indicates compounds whose action is Cys-347-dependent are not robustly predicted by these first-pass computational filters as most of the molecules suspected of covalent interaction with the GLP-1R have <100 demerits (non-covalent GLP-1R PAMs are indicated in the *box* in Fig. 7*B* with activity ratios of wild-type/C347A ≈1). Moreover, analysis of the pharmacological selectivity of Cys-347-dependent compounds indicated these compounds were generally selective and did not exhibit a pan-assay active profile as has been described for some known problematic chemotypes (Fig. 7*C*) (47, 48).

## Discussion

Several therapeutic peptides that mimic the binding and receptor activation mechanism of GLP-1 have been approved and registered for the treatment of type 2 diabetes mellitus. Fundamental insights into the complex ligand binding process at the orthosteric site to induce GLP-1R signaling have been gained by use of various experimental approaches. X-ray crystallography studies of the GLP-1R ECD in complex with peptide ligand have revealed how the carboxyl-terminal α-helical region of GLP-1 positions within the binding cleft of the ECD (49). Furthermore, photo-affinity labeling has enabled determining residues that position in close proximity between the ligand and the full-length receptor (50, 51). Complementary GLP-1R chimera and mutagenesis studies have proposed additional key residues that may further define the core peptide-binding pocket (52, 53). Together, these data have informed and refined molecular modeling of the peptide-bound GLP-1R.

As an alternative approach, the studies presented here were undertaken to investigate an allosteric mechanism for activation of GLP-1R. Identifying and understanding mechanisms that modulate the GLP-1R may enable strategies to discover and develop new therapeutic agents, especially at receptor sites distinct from the orthosteric pocket that binds non-peptide low molecular weight compounds. BETP is an electrophilic small molecule that enhances GLP-1R signaling (19, 22) by binding covalently to Cys-347 within the third intracellular loop of the receptor (26). In general, proteins containing free nucleophilic amino acids undergo modification upon being in close proximity to reactive compounds, which often results in widespread irreversibly bound small molecules in cells and tissues. A significant finding from the studies performed herein is that the insulinotropic activity of BETP was demonstrated to be specific for the GLP-1R in *ex vivo* pancreatic islet assays; here, BETP required the presence of the GLP-1R, high glucose, and the peptide ligand to induce insulin secretion. The findings from these islet culture experiments are important because the results provide physiologically relevant evidence that modulating intracellular regions of the GLP-1R, especially within the vicinity of the third intracellular loop, may be a mechanism to regulate receptor signaling.

Interestingly, dynamic simulations of a variant GLP-1R using a homology model (54) also supports the potential importance of the third intracellular loop of the GLP-1R. In efforts aimed at identifying coding variants that underlie susceptibility for developing type 2 diabetes mellitus, an A316T substitution in the GLP-1R was found to associate with lower fasting blood glucose concentrations but higher glucose levels 2 h following a glucose challenge (55). Position 316 is located in transmembrane domain (TMD) 5, and the modeling shows the threonine variant disrupts hydrogen bonding between residues in TMD5 and TMD6 (55). These changes predict a shift of TMD5 toward the cytoplasm and movement of TMD6 outward, altering the position of the third intracellular loop (55). The possibility that a subtle shift in loop three by a variant may alter GLP-1R function is consistent with the ability of BETP to form a small adduct with Cys-347 in this region to alter receptor signaling.

The capacity of BETP to modulate GLP-1R signaling by covalently modifying the GLP-1R suggests this mechanism could be exploited therapeutically. In addition to BETP, this report shows that several structurally diverse compounds can also covalently modify the GLP-1R to enhance activation by GLP-1(9–36)-NH_2_. The newly discovered compounds that potentiate GLP-1R signaling require Cys-347 for activity, and unfortunately, these molecules are also electrophilic to GSH, which may indicate the critical pharmacological feature for recognizing the GLP-1R is the intrinsic electrophilic nature of the compounds. Although covalent molecules have been developed as therapeutic agents (56), if there is not a canonical binding pocket present around Cys-347 (and only reactive compounds interact with this amino acid), it is unlikely that drug-like molecules can be developed due to pharmacokinetic and safety liabilities (57). Classical approaches for the evaluation of covalent enzyme inhibitors classify compounds as affinity labels (reactivity-driven) or quiescent affinity labels (reversible binding-driven) (58, 59). Only the latter have drug-like potential. By examining the calculated rate constants for GSH modification of electrophiles from this study, they appear to be in the range of <100 m^−1^ s^−1^ values typical of affinity labels. Subsequent studies could be directed toward a quantitative understanding of structure-activity relationships of GLP-1 PAMs and inherent electrophilicity toward GLP-1R. This would require either a sensitive kinetic assay to measure time dependence of compound action or a direct binding assay. At this point, we do not have robust assay systems capable of resolving GLP-1R-specific binding from the midst of high nonspecific binding for [^3^H]BETP. Generation of an effective allosteric GLP-1R probe with less electrophilicity would be useful to enable these studies. It remains uncertain whether allosteric modulators of the GLP-1R that function via the covalent mechanism can be identified that are stable in the presence of other nucleophiles.

The overall results from these studies highlight the nucleophilic nature of the GLP-1R, which may influence the direction of future work in this area. For example, this characteristic should be considered when pursuing new screening campaigns for identifying GLP-1R activators. An early understanding of the mechanism of action can avoid the prosecution of promiscuous compounds (60). Many electrophiles can be recognized by features of their chemical structure; however, assays to assess the degree of electrophilicity are useful for conclusively determining the reactivity of some chemotypes. Our results suggest though that although various assays can be used to filter out reactive molecules, C347A GLP-1R is a critical assay for identifying compounds that covalently modify the GLP-1R to enhance its activity. For instance, it is possible that active compounds could contain independent electrophilic and GLP-1R-binding pharmacophores. Such compounds would be excluded from follow-up if triage was exclusively based on chemical reactivity. In our view, it will be important to determine whether several recently reported GLP-1R PAMs require Cys-347 for activity (61–63). Moreover, our data clearly show that the types of electrophiles that modulate the GLP-1R can be somewhat cryptic and are not generally well predicted by computational approaches, prior learning, and general compound promiscuity information. Our data also indicate that a substantial fraction of GLP-1R allosteric modulators identified by screening may act via covalent modification of Cys-347.

The nucleophilic capacity of the GLP-1R may be exploited experimentally to stabilize the GLP-1R in biophysical studies. It is clear that adding steric bulk around the vicinity of Cys-347 produces changes in the protein conformation that promote receptor signaling. Importantly, some of the newly identified scaffolds that modulate the GLP-1R in a Cys-347-dependent manner may possess physicochemical properties suitable for various crystallography conditions.

## Author Contributions

J. F. and O. C. designed, performed, and analyzed the islet insulin secretion studies. A. D. S. and C. S. performed and analyzed the GTPγS studies. A. D. S. and D. B. W. designed, performed, and analyzed the cAMP accumulation studies. A. M. developed and performed the GSH conjugation assays using LC/MS detection. F. S. W. developed, performed, and analyzed the fluorescein-maleimide competition assays. A. B. B., F. S. W., and K. W. S. conceived, coordinated the study, designed the experiments, analyzed the data, and wrote the paper. All authors reviewed the results and approved the final version of the manuscript.

## Footnotes

2 The abbreviations used are:

GPCR: G protein-coupled receptor
GLP-1R: glucagon-like peptide-1 receptor
TMD: transmembrane domain
PAM: positive allosteric modulator
GTPγS: guanosine 5′-3-*O*-(thio)triphosphate
ECD: ectodomain
th-BETP: 4-(3-benzyloxyphenyl)-2-ethylsulfanyl-6-(trifluoromethyl)pyrimidine
ANOVA: analysis of variance
ACN: acetonitrile
FA: Formic acid
Z: benzyloxycarbonyl
AMC: amido-4-methylcoumarin
CatL: cathepsin L
BETP: 4-(3-benzyloxyphenyl)-2-ethylsulfinyl-6-(trifluoromethyl)pyrimidine
EBSS: Earle's balanced salt solution buffer.
